# 肺鳞状细胞癌视网膜转移1例

**DOI:** 10.3779/j.issn.1009-3419.2017.09.11

**Published:** 2017-09-20

**Authors:** 金霞 刘, 茜倩 高, 珉丹 孙

**Affiliations:** 130000 长春，吉林大学第一医院肾病内科 Department of Nephrosis Internal Medicine, the First Hospital of Jilin University, Changchun 130000, China

**Keywords:** 视网膜转移, 肺肿瘤, 化疗, Retinal metastasis, Lung neoplasms, Chemotherapy

## Abstract

肺癌是临床上常见的恶性肿瘤，发病率和死亡率居肿瘤首位，严重威胁人类的健康。由于早期缺乏有效的、特异性强的筛查方法，多数患者发现时已处于中晚期，常合并骨、脑、肝、肾上腺等部位的转移。通常，患者多因原发病灶、扩散转移或副肿瘤综合征等引起临床症状而就诊。对由于远处转移相关症状作为首发表现就诊的患者，诊断是一个非常大的挑战。视网膜转移作为肺鳞癌的首发症状极其罕见。本文回顾性分析了我院收治的一例肺鳞癌患者的诊治经过，以眼部症状为首发表现，基于临床表现、影像学检查和手术病理诊断，给予患者手术、化疗等多学科综合治疗，目前短期预后良好，随访观察中。我们总结了本病例诊疗过程的特点，为临床医生提供经验。同时复习肿瘤眼内转移的相关文献，为我们对于肺癌罕见表现的深入了解提供了一个窗口。

## Abstract (en)

Lung cancer is a common malignant tumor in clinic. Morbidity and mortality are the first place in cancer, which seriously threaten the health of human beings. Due to the lack of effective and specific screening methods, most patients were found already at an advanced stage, and often combined with bone, brain, liver, adrenal and other parts of the metastasis. Symptoms of patient resulted from the primary tumor, metastatic spread, or paraneoplastic syndrome commonly cause the medical attention. For the syndrome related to the distant metastatic spread as the first manifestation, diagnosis would be a challenge. Retinal metastasis is very rare as the first symptom of squamous cell carcinoma of the lung. This paper reviewed a case, in which a patient developed the retinal metastasis as the inital presentation. After systemic evaluation, our patient was managed in multidisciplinary treatments, including surgery and chemotherapy. Now, the patient has a good short-term prognosis and is still in fellow-up condition. Later, we summarized the characteristics of the diagnosis and treatment of this case and provided experience for clinicians. At the same time, reviewing the relevant literature on tumor metastasis provides a window for our understanding of the rare manifestations of lung cancer.

## 1 病例资料

患者，女，62岁，因“左眼疼痛、视力模糊半个月，发现右肺占位10天”于2016年12月15日入我院胸外科。患者半个月前活动时突然出现左眼胀痛，进行性加重，后出现视物模糊，视野残缺，就诊于外院，行视力、眼部超声、胸片等检查提示球内占位、右肺下叶占位，为进一步诊治就诊于我院。既往：2年前因多发室性早搏于我院心内科行射频消融术，术后心率平稳，在60-100次/分之间。家族史无特殊。辅助检查：（2016.12.05）检查视力左眼0.1，右眼0.8。欧堡全景200T×激光扫描检眼镜检查（Optos-Panoramic 200 scanning laser ophthalmoscope, Optomap 200T×）示左眼眼底颞上方视网膜见黄色渗出（图 1A），右眼无异常。左眼超声：左眼玻璃体内可见弱中点状回声，眼底球壁可见中低实性团状回声，表面回声增强，内回声均匀，边界清，CDFI显示光团内，可见丰富血流信号。影像诊断球内占位。视野检查：左眼鼻下方视野缺损。头部核磁未见明显异常。胸片提示右肺下叶占位。入院后行正电子发射断层显像/X线计算机体层成像检查（positron emission tomography computed tomography, PET-CT）示右肺下叶背段高代谢团块，大小3.3 cm×2.8 cm×2.4 cm，考虑中心型肺癌；左侧眼底局限性增厚，代谢不高（图 2）。进一步行支气管镜活检术，病理提示：右下背段新生物非小细胞肺癌，倾向鳞状细胞癌。联合眼科、放射线科等多学科会诊，全面评估患者病情，考虑患者眼部病变来自肺部转移可能性大，可先行手术切除肺部原发病灶。遂于2017年12月20日于我院胸外科行右肺中下叶切除及淋巴结廓清术，过程顺利。术后病理提示：右肺下叶鳞癌T2aN1 IIa期，免疫组化CK5/6（+）、CK7（-）、Ki-67（+40%）、P63（+）、TTF-1（-）。2017年1月22日于眼科复诊：左眼视力进行性下降，仅有光感。Optomap 200T×示左眼眼底颞上方视网膜黄色渗出范围较前明显扩大（图 1B）。眼底镜检查提示颞上方视网膜见大片白色渗出，边缘不规则（图 3A）。光学相关断层扫描（optical coherence tomography, OCT）提示黄斑区视网膜局限性隆起增厚（图 4A）。建议患者行细针穿刺活检术明确病理诊断，考虑到相关风险，患者及家属拒绝行活检术。再次联合眼科、放射线等多学科会诊，明确患者后期治疗方案。2017年2月6日于我院肿瘤科行[吉西他滨+顺铂]（吉西他滨1.6 g d1、d8静点，顺铂40 mg d2-d4静点）联合化疗。1疗程后复查左侧视力上升至0.2-0.3，Optomap 200T×示病灶范围较前缩小（图 1C）。2疗程后复查左侧视力上升至0.6-0.7，Optomap 200T×示病灶范围较前再次缩小（图 1D），眼底镜检查提示颞上方视网膜白色渗出范围亦较前变小（图 3B），OCT提示黄斑中心凹形态大致恢复正常（图 4B）。2017年4月19日，患者4疗程术后辅助化疗结束。患者继续于外院行眼部随诊，电话随访，患者目前状态良好，复查左侧视力现恢复至0.8，全眼底照相病灶基本消失，暂无需手术、激光等进一步治疗，继续观察随访中。

**1:**
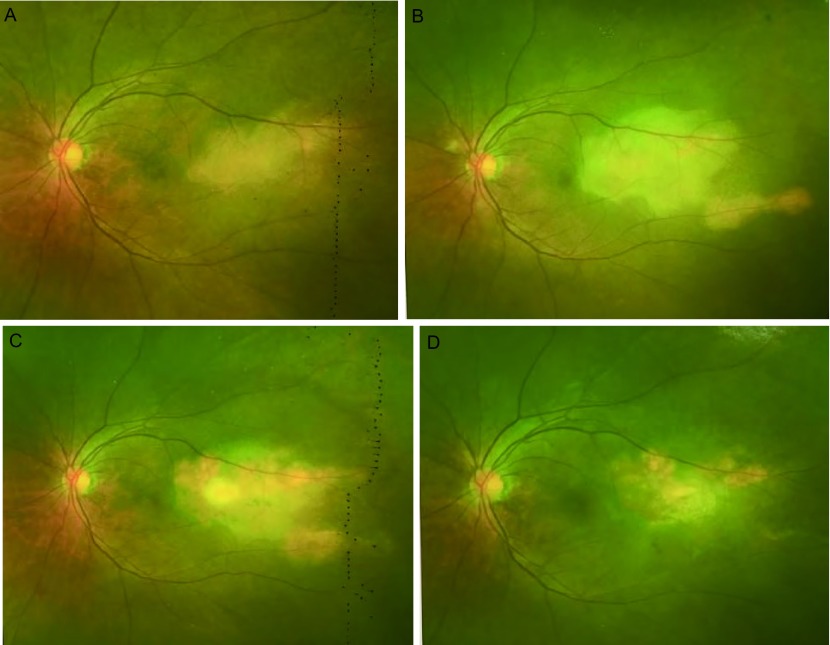
62岁女性患者，诊断为肺鳞状细胞癌合并视网膜转移，行手术切除肺部原发病灶，术后应用吉西他滨加顺铂辅助化疗4疗程。诊疗过程中Optomap 200T×图像变化。A（2016.12.05）：发病初期，可见左眼眼底颞上方视网膜见黄色渗出。B（2017.01.22）：肺癌术后，化疗前，眼部疾病进展，病灶范围较前增大。C（2017.02.23）：行吉西他滨+顺铂1疗程化疗后，病灶范围略缩小。D（2017.03.17）：行吉西他滨+顺铂2疗程化疗后，病灶范围较化疗前明显缩小。 A 62 years old woman, who was diagnosed aspulmonary squamous cell carcinoma with retinal metastasis, underwent surgical resection of the primary lung lesion and 4 courses adjuvant chemotherapy of gemcitabine plus cisplatin. Follwoing are the changes of Optomap and 200Tx images during diagnosis and treatment. A (2016.12.05): Initial stage of disease, yellow exudate was seen in the retina above the left eye. B (2017.01.22): After Lung squamous carcinoma operation, before chemotherapy, the disease was progressed and the range of lesion increased. C (2017.02.23): After 1 chemotherapy course of gemcitabine+cisplatin, the lesion contracted slightly. D (2017.03.17): After 2 chemotherapy course of gemcitabine+cisplatin, the lesion was significantly smaller than before chemotherapy.

**2:**
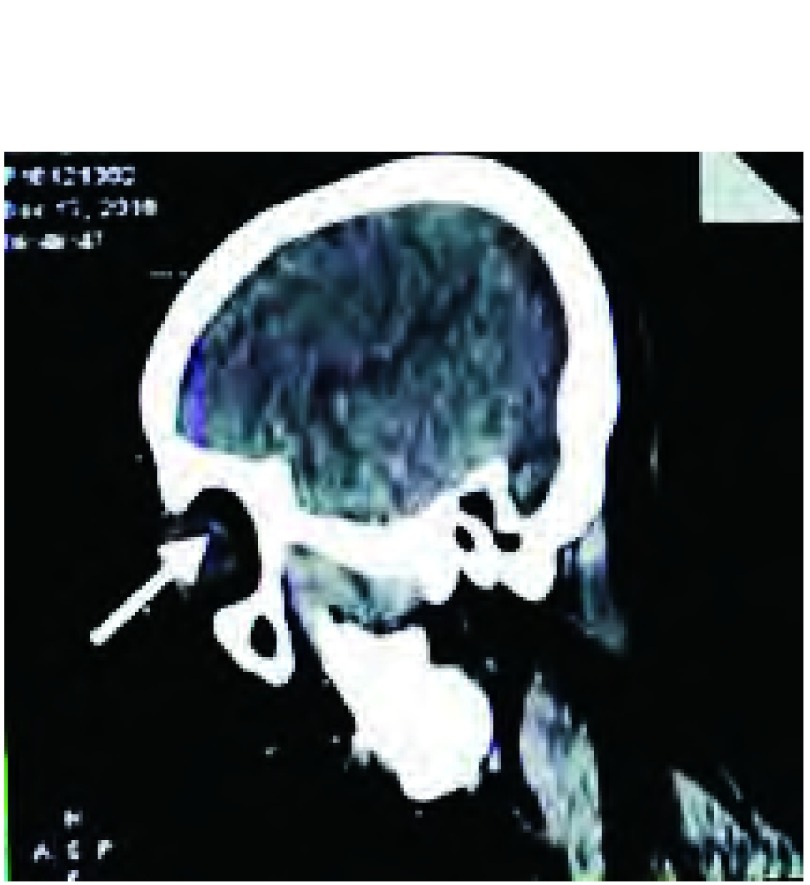
患者发病初期PET-CT检查显示左侧眼底局限性增厚，代谢不高。 At the onset of the disease, PET-CT showed a Localized thickening of the left fundus with low metabolism.

**3:**
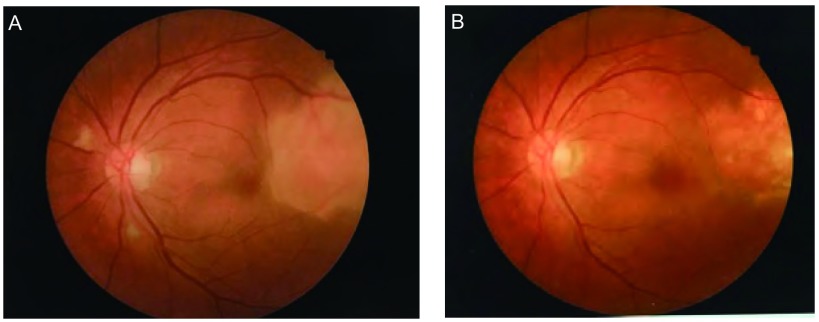
患者治疗过程中眼底镜检查图像变化。A（2017.1.22）：肺癌术后，化疗前，可见颞上方视网膜见大片白色渗出，边缘不规则。B（2017.3.17）：行吉西他滨+顺铂2疗程化疗后，渗出范围较化疗前明显缩小。 Changes in Funduscopy images during the treatment. A (2017.1.22): After lung squamous carcinoma operation, before chemotherapy, white exudate was seen in the retina above the left eye with irregular margins. B (2017.3.17): After 2 chemotherapy course of gemcitabine+cisplatin, the lesion was significantly smaller than before chemotherapy.

**4:**
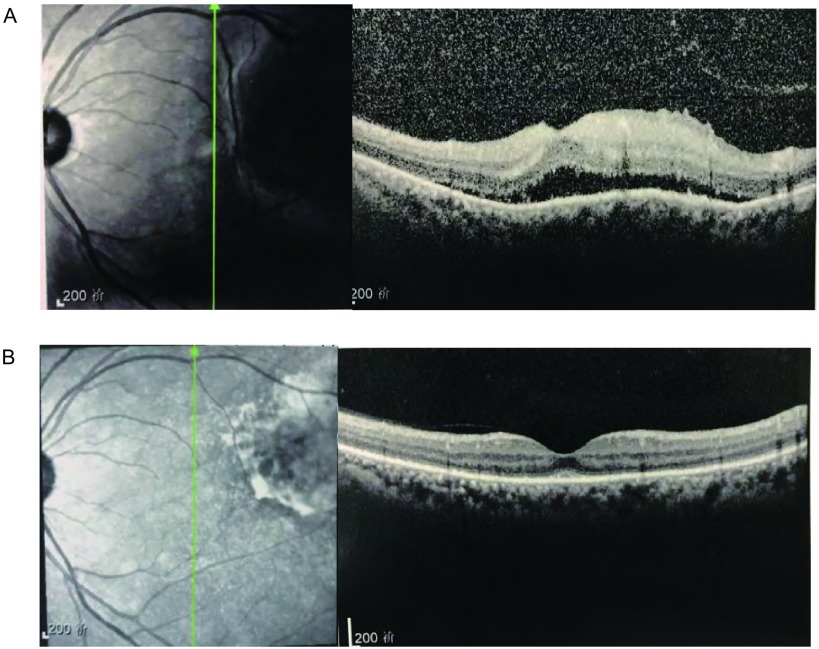
患者治疗过程中OCT图像变化。A（2017.1.22）：肺癌术后，化疗前，OCT检查示黄斑区视网膜局限性隆起增厚。B（2017.3.17）：行吉西他滨+顺铂2疗程化疗后，OCT检查示黄斑中心凹形态大致恢复正常。 Optical coherencetomography (OCT): A (2017.1.22): After lung squamous carcinoma operation, before chemotherapy, OCT showed localized thickening at the fovea. B (2017.3.17): After 2 chemotherapy course of gemcitabine+cisplatin, OCT showed that the shape of fovea was approximately normal.

## 2 讨论

肺癌是我国乃至全球发病率和死亡率最高的恶性肿瘤。近年来，该疾病的发病率在我国呈显著上升趋势。虽然世界各国都投入了大量的人力和物力，肺癌的诊治依然没有质的突进。多数患者发现时已处于中晚期，常合并骨、脑、肝、肾上腺等部位的转移。眼睛作为人体一种特殊的视觉器官，主要由眼动脉供血，眼动脉与颈内动脉呈直角，血流中的肿瘤栓子等由于血流速度关系，往往容易停留在颅内，而不易经眼动脉进入眼内，故眼转移瘤十分少见，且常晚发于其他部位。但转移瘤仍是眼部最常见的恶性肿瘤。最早的眼部转移瘤由Horner报告于1864年，是一例肺癌眼眶转移患者^[1]^。眼部转移最常见的部位是葡萄膜^[2]^。脉络膜由于血管丰富最常受影响（89%），其次是虹膜（9%）和睫状体（2%）^[3]^。目前文献对视网膜转移癌的报道极少，以眼部症状为首发表现者更少。多数视网膜转移癌来自于皮肤黑色素瘤，小部分来自于乳腺癌和肺癌^[4]^。既往报道的31例视网膜转移患者，其中有9例原发病灶在肺部^[5-6]^。Su等^[7]^调查了1991年至2005年被诊断为肺癌的8484余例患者，其中有16例合并眼部转移，仅有2例合并视网膜转移，肺癌眼部转移瘤发病率小于1%。原发肺癌眼部转移患者最常见的组织类型为腺癌，鳞癌少见^[8]^。考虑可能与原发肿瘤的转移特点有关，腺癌以血液循环转移途径为主，鳞癌以淋巴管转移为主，由于眼部缺少淋巴管，故眼部转移主要通过血行转移。本例患者为肺鳞状细胞癌合并视网膜转移，以眼部症状为首发表现，相对更少见。

眼部转移瘤临床表现多种多样，本例患者为左眼单发病灶，以眼部胀痛为最初表现，随后出现视物模糊、视力下降甚至失明。既往报道中提到的其他眼部症状有飞蚊症、畏光、复视、眼球突出、视网膜脱离等。眼部病灶多为单侧单发病灶，亦有双侧多发转移灶报道。Shields等^[9]^报告的8例视网膜转移患者有7例为单侧单发。部分学者提出左眼转移比右眼转移几率高，可能与血管与解剖学上左侧颈总动脉直接从主动脉弓分支，而右颈总动脉由头臂干分支而来，故与癌细胞更容易到达左侧有关，但多种因素都会对此造成影响，所以需要更多的统计数据来证实。

眼部转移瘤的诊断主要依靠临床症状和体征、眼科专科检查、影像学检查如眼部MRI和眼部CT等，病理学检查依然是最准确的诊断方法。因本例患者视网膜转移病灶靠近黄斑区，穿刺活检风险大，有极大可能误伤黄斑区造成永久性视野缺损或失明，故未行视网膜转移病灶活检明确病理诊断。本例患者临床诊断为肺鳞状细胞癌视网膜转移有一定挑战性，尤其是在没有转移病灶的病理活检结果作为支持时。关于视网膜病变的相关鉴别诊断亦被考虑在内，如视网膜母细胞瘤、视网膜炎症、视网膜血管阻塞、视网膜变性及营养不良等。对于本例患者的视网膜转移病灶，在术后辅助化疗过程中，在未针对其应用相应抗生素、激素等特殊药物治疗情况下，随着化疗的进行，眼部症状好转，转移病灶缩小，支持视网膜转移癌的诊断。

目前对于合并眼部转移瘤的患者主要采取姑息治疗，眼转移瘤的发生预示眼部以外的其他器官也可能存在转移性病灶。治疗的目的是在控制原发病灶和转移灶进展的同时，保护部分视觉功能，减少患者痛苦，提高生存质量。治疗措施主要有手术（眼球摘除、减瘤术）、化疗、放疗、靶向治疗及观察支持治疗等。对于眼部转移灶来说，放射性治疗是首选^[10]^。但作为全身治疗手段，化疗是一种主要的方式。眼部转移瘤对化疗的敏感性取决于肿瘤原发灶的病理类型，应依据原发灶病理特点，选择合适的化疗药物。有文献指出，多数患者经过个体化治疗，眼部转移瘤可得到局部控制，短期视力恢复预后较好，但是总体预后依然很差，多在2个月到2年内死于肿瘤其他部位转移^[11, 12]^。通常认为眼部转移预示着肿瘤的终末期，多数合并除眼部病灶以外的其他器官转移^[9]^。Su等^[7]^报道的16例肺癌眼转移患者，有13例患者合并除眼部以外的远处器官转移。眼部肿瘤转移预示着癌细胞已进入血液循环，突破血脑屏障，但同时也会破坏血脑屏障，有神经系统及其他多系统转移的风险，这种多系统转移相互影响常可导致患者死亡。在合并视网膜转移的恶性肿瘤患者中，视觉症状发作后的中位生存期约为9个月^[13]^。Shields等^[9]^报告的8例肺癌合并视网膜转移患者，确定眼部转移灶后死亡时间的中位数为1个月，其中5个患者在确诊后1个月内死亡。Mack等^[13]^分析了的20例皮肤黑色素瘤视网膜转移患者的生存时间，患者均在视觉症状出现后的2周到5年内死亡。本例患者肺部原发病灶为IIa期，无视网膜以外的远处转移灶，已对肺部原发灶行手术切除，且行术后辅助化疗，眼部症状在1疗程化疗后好转，4疗程化疗后视网膜转移病灶基本消失，故本例患者短期预后相对较好，但长期预后仍需观察随访。

对于肺癌合并眼部转移瘤患者的诊断治疗需眼科、放射线科、呼吸科等多学科的共同努力。我们强调对于肺癌患者出现眼部症状时眼科专科检查的重要性，对发现眼部异常表现时需警惕眼部转移瘤的可能，做到早发现、早诊断和早治疗，提高患者的生活质量，改善预后。
